# Investigation of two norovirus outbreaks linked to drinking water contaminated with multiple GII strains in a rural county—Chongqing, China, 2021

**DOI:** 10.3389/fpubh.2023.1259584

**Published:** 2023-12-14

**Authors:** Tingting Li, Jingyao Peng, Qin Li, Baisong Li, Yi Yuan, Chuan Yang, Di Yang, Wenge Tang, Li Qi

**Affiliations:** ^1^Chongqing Municipal Center for Disease Control and Prevention, Chongqing, China; ^2^Chongqing Municipal Key Laboratory for High Pathogenic Microbes, Chongqing, China; ^3^Xiushan County Center for Disease Control and Prevention, Chongqing, China

**Keywords:** norovirus, GII strains, outbreak, drinking water, rural county, China

## Abstract

**Backgrounds:**

Norovirus is leading cause of non-bacterial gastroenteritis outbreaks globally, characterized by different strains prevalent in different countries and regions.

**Methods:**

Cases were defined as individuals experiencing diarrhea ≥3 times/24 h, and/or vomiting ≥2 times/24 h in two villages between January 28 and February 9, 2021. Investigations were conducted to identify causes. Cases were interviewed using a standardized in-person form to collect data on potential risk factors. A retrospective cohort study was conducted to investigate the role of the spring water supply as the outbreak source. Residents from neighboring villages with different water sources served as the unexposed population. Stool specimens, rectal swabs, and water samples were tested using quantitative real-time Polymerase Chain Reaction, with subsequent sequencing performed on pathogen-positive specimens.

**Results:**

Village-specific attack rates were 21.93% (123/561) and 26.99% (88/326), respectively. Evidence from both epidemiological and laboratory tests was consistent. Drinking spring water was statistically associated with the two outbreaks (RR = 41.8 and 79.2, respectively). In both outbreaks, stool specimens, rectal swabs, and water samples tested positive for norovirus. Specifically, GII.2 (P16) and GII.17 (P17) were identified in outbreak A, and GII.4 Sydney (P16) and GII.1 (P16) in outbreak B.

**Conclusion:**

These two independent gastroenteritis outbreaks share similarities, both being linked to norovirus GII strains. The contaminated spring drinking water was identified as the probable source and was promptly closed and subjected to disinfection procedures. These findings reinforce the importance of implementing sanitation and environmental disinfection measures in rural areas, especially during the periods of increased rainfall.

## 1 Introduction

Human norovirus is one of the most prevalent pathogens causing acute gastroenteritis worldwide (1). It is extremely contagious, with an estimated infectious dose of as minimal as 18 viral particles (2). Norovirus can be transmitted through various routes, including waterborne, foodborne, or person-to-person contact. Multiple transmission routes involving in one norovirus outbreak is not rare (3–7). The transmission route in a norovirus outbreak varies based on the setting and geographical region. In China, 93% of the norovirus outbreaks occurred in schools, the dominant transmission route was person-to-person contact, accounting for 63% of all norovirus outbreaks reported to the National Public Health Emergency Event Surveillance System from 2014 to 2017, and waterborne transimission only accounted for 3.4% (8).

In China, the GII (genogroup II) was mostly detected genogroup in norovirus outbreaks. From October 2016 to September 2018, the most commonly reported strain in norovirus outbreaks was GII.2 (P16) (9). From 2014 to 2018, multiple GII strains were identified in norovirus outbreaks, and the dominant GII strains showed temporal variations in different cities (10–12). For instance, in Jiangsu, China, the GII.2 (P16) strain was mostly identified in norovirus outbreaks from January 2015 to December 2018 (10). In Huzhou, China, the predominant strains in norovirus outbreaks changed from GII.4 variants to GII.17 (P17) in 2014–2015, followed by GII.3 (P12) in 2015–2016, and later GII.2 (P16) in 2016–2018 (11). In Guangdong, China, from 2013 to November 30, 2017, the majority of reported norovirus outbreaks were typed as GII, with GII.17 strain and GII.2 strain peaked during winter seasons of 2014–2015 and 2016–2017, respectively (12).

In 2011, the first norovirus infectious diarrhea case was reported in Chongqing, China. From 2011 to 2016, a total of 121 norovirus epidemic events (clustered epidemics and outbreaks) were reported in Chongqing. These events involved 1,637 cases, with no reported fatalities. Nearly all epidemic events occurred in schools, and 80% were transmitted through person-to-person contact (13). Among the 121 epidemic events, the GII.2 strain was identified as the predominat pathogen through sequencing analysis (13). The data from the National Surveillance System for Disease Control and Prevention indicated that, from 2017 to 2022, an average of approximately 40 norovirus outbreaks were reported annually in Chongqing.

Many norovirus outbreaks and clustered epidemics are reported in China annually. Previous studies have predominantly focused on the long-term epidemiological characteristics of these outbreaks in China or the distribution of norovirus genotypes (9, 14). However, relatively few studies have conducted on norovirus outbreak responses. In early February 2021, a county in Chongqing confronted a sudden surge of cases, with reported dozens of villagers in two separate villages experiencing diarrhea and vomiting for unknown reasons. In response to these outbreaks, investigations were conducted. This paper aims to elucidate the two gastroenteritis outbreaks, identify the causes, detect possible associations between the two outbreaks, and provide scientific information for preventing further transmission.

## 2 Methods

### 2.1 Case definition

In early 2021, two separate gastroenteritis outbreaks occurred in county A were reported repeatedly to Chongqing Municipal Center for Disease Control and Prevention (CDC). To control outbreaks, epidemiological investigations were conducted immediately by an investigation team including epidemiologists, physicians, and laboratory technicians.

For both outbreak A and outbreak B, a probable case was defined as an individual who had experienced diarrhea ≥3 times per 24 h, and/or vomiting ≥2 times per 24 h from January 28 to February 6 and from February 2 to February 9, 2021, respectively.

A laboratory-confirmed case in both outbreaks was defined as a probable case in which a stool specimen, an rectal swab, or a vomit sample was tested positive for norovirus using quantitative real-time polymerase chain reaction (RT-PCR).

### 2.2 Case finding

Case finding was conducted in each villager’s home and local village clinics. Villagers with symptoms of diarrhea and/or vomiting within the previous 3 days during each outbreak were investigated for case confirmation. To enhance the accuracy of case identification, doctors in the local village clinics were interviewed to obtain clinical symptoms, onset time, and other related information of treated patients in recent days during each outbreak. Cases were interviewed in person with a standardized form to collect clinical and epidemiology data. This included name, age, gender, occupation, address, time of onset, symptoms (diarrhea, vomiting, stomach cramping, abdominal distension, etc.), whether to use the centralized water supply and other related information.

### 2.3 Environmental investigation

We inspected the environmental hygiene regarding water sources and the living environments of the cases. For the water sources of the two villages, their coverage of residents, disinfection facilities and frequency, and the overall sanitation were examined carefully. For the cases’ homes in each outbreak, investigations regarding water used for drinking and cooking, and water purifiers (manufacture of water bands and equipment) were conducted. Other public activities related to water use, such as banquets, gatherings, and washing clothes in the public pool were also investigated.

### 2.4 Retrospective cohort study

Evidence from the descriptive epidemiology investigation and environmental investigation indicated that water source was the most probable risk factor for both outbreaks. To verify this hypothesis, a retrospective cohort study was conducted, using residents from incriminated villages and neighbor villagers with different water sources to identify risk factors for each outbreak.

### 2.5 Sample collection and preprocessing

Stool specimens, rectal swabs, and vomit samples from cases were collected. Water samples from the water sources, reservoirs (containing water from different water sources), and the homes of the cases were also collected.

For the nucleic acid extraction from water samples, we used 8 mL of lysis buffer (QVL Lysis Buffer) to lyse 2 mL of water sample according to the ratio in the kit (OMEGA E.Z.N.A Viral RNA Kit) for 10 min, added 6 mL of absolute ethanol, and mixed well. The above liquid was filtered through a cellulose acetate membrane filter column (HiBind RNA Mini Column) using a suction filtration pump, and then the relevant operations were carried out according to the kit steps, and the final obtained RNA was equivalent to concentrating about 14 times.

For the nucleic acid extraction from cases’ samples, we used an automatic nucleic acid extraction instrument (SSNP-3000A) and matched supporting reagents. Rectal swabs were directly added with 200 uL reagent samples for nucleic acid extraction, about 3 g of stool specimens were added with 10 mL phosphate-buffered solution, thoroughly mixed and centrifuged, and 200 uL of supernatants were taken for nucleic acid extraction.

### 2.6 Laboratory detection

The samples were tested twice using a commercial RT-PCR kit (Norovirus GI/GII Nucleic Acid Detection Kit from Jiangsu Bioperfectus Technologies Co., Ltd.).

For the nucleic acid of the samples tested positive for norovirus by RT-PCR, part of the region including the polymerase region and capsid region was amplified using the Prime Script One-Step RT-PCR Kit Ver.2. The amplified product was sent to the Beijing Genomics institution for sequencing.

The successfully sequenced nucleic acid sequences were spliced, edited, and organized by DNA STAR software, and the nucleic acid sequences were compared on the NCBI website,^1^ and the sequence with the highest homology known sequence types was used as typing results. Phylogenetic analysis was performed using MEGA 7 software.^2^ All reference sequences were downloaded from Genbank, sequence alignment was performed using Clustalw, the phylogenetic tree was constructed by the Neighbor-Joining method, and bootstrap was used to test 1,000 times repeatedly.

### 2.7 Statistical analysis

The demographic characteristics and clinical symptoms of cases were displayed with proportions. Chi-square tests or Wilcoxon rank sum tests were conducted to compare differences in attack rates, gender, age, and occupation among cases between the cases in the two outbreaks. A *p*-value of less than 0.05 (two-tailed) was determined to be statistically significant. All statistical analyses were performed using Excel and Epi info (version 7.2.5.0). ArcGIS software was used to visualize the locations of the two outbreaks.

## 3 Results

### 3.1 Epidemiological characteristics

Two norovirus outbreaks occurred in two separate villages of county A in Chongqing, China, in late January and early February of 2021. The two villages are located 34 miles apart and there were no common activities recently among villagers from the two villages. In outbreak A and outbreak B, 561 villagers and 328 villagers shared the same spring water supply, respectively (Supplementary Figure S1).

A total of 123 cases (including nine laboratory-confirmed cases) and 88 cases (including 37 laboratory-confirmed cases) were reported in the two outbreaks, with attack rates of 21.93% (123/561) and 26.99% (88/326). The median age was 47.0/41.5 years, males accounted for 52.85%/51.14%, and farmers accounted for 85.37%/78.41%, with no statistically significant differences in age (*p* = 0.427), gender (*χ*^2^ = 0.06, *p* = 0.806), occupation (*χ*^2^ = 6.61, *p* = 0.359), and attack rates (*χ*^2^ = 2.92, *p* = 0.087) of the two outbreaks. Seventy-four household cluster cases were identified in outbreak A and 39 in outbreak B. The major clinical symptom of cases was diarrhea (95.93%) and vomiting (59.09%) in two outbreaks, respectively. Detailed information is shown in Table 1.

**Table 1:** Epidemiological characteristics of the two norovirus outbreaks occurred in early 2021.

Characteristics	Outbreak A (*n* = 123)	Outbreak B (*n* = 88)	*χ*2	*p*
Demographic characters				
Age (range, median)	2–84 yrs., 47 yrs	10 mons–83 yrs., 41.5 yrs		0.427^c^
Gender (male, %)	65, 52.85	45, 51.14	0.06	0.806
Occupation (peasant, %)	105, 85.37	69, 78.41	6.61	0.359
Clinical symptoms (*n*, %)				
Diarrhea^a^	118, 95.93	45, 51.14		
Vomiting^b^	46, 37.40	52, 59.09		
Stomach cramping	59, 47.97	—		
Abdominal distension	—	27, 30.68		
Transmission route	Waterborne, person-to-person	Waterborne, person-to-person		
Start date of the outbreak	January 31st	February 5th		
End date of the outbreak	February 6th	February 9th		
Duration (days)	7	5		
Number of cases	123	88		
Number of persons at risk	561	326		
Attack rate	21.93	26.99	2.92	0.087
Confirmed case	9	32		
Household cluster case	74	39		

yrs, years; mons, months; “—” no cases were reported.

a Diarrhea ≥3 times per 24 h.

b Vomiting ≥2 times per 24 h.

c *p*-value obtained from Wilcoxon rank sum test.

**Figure 1:**
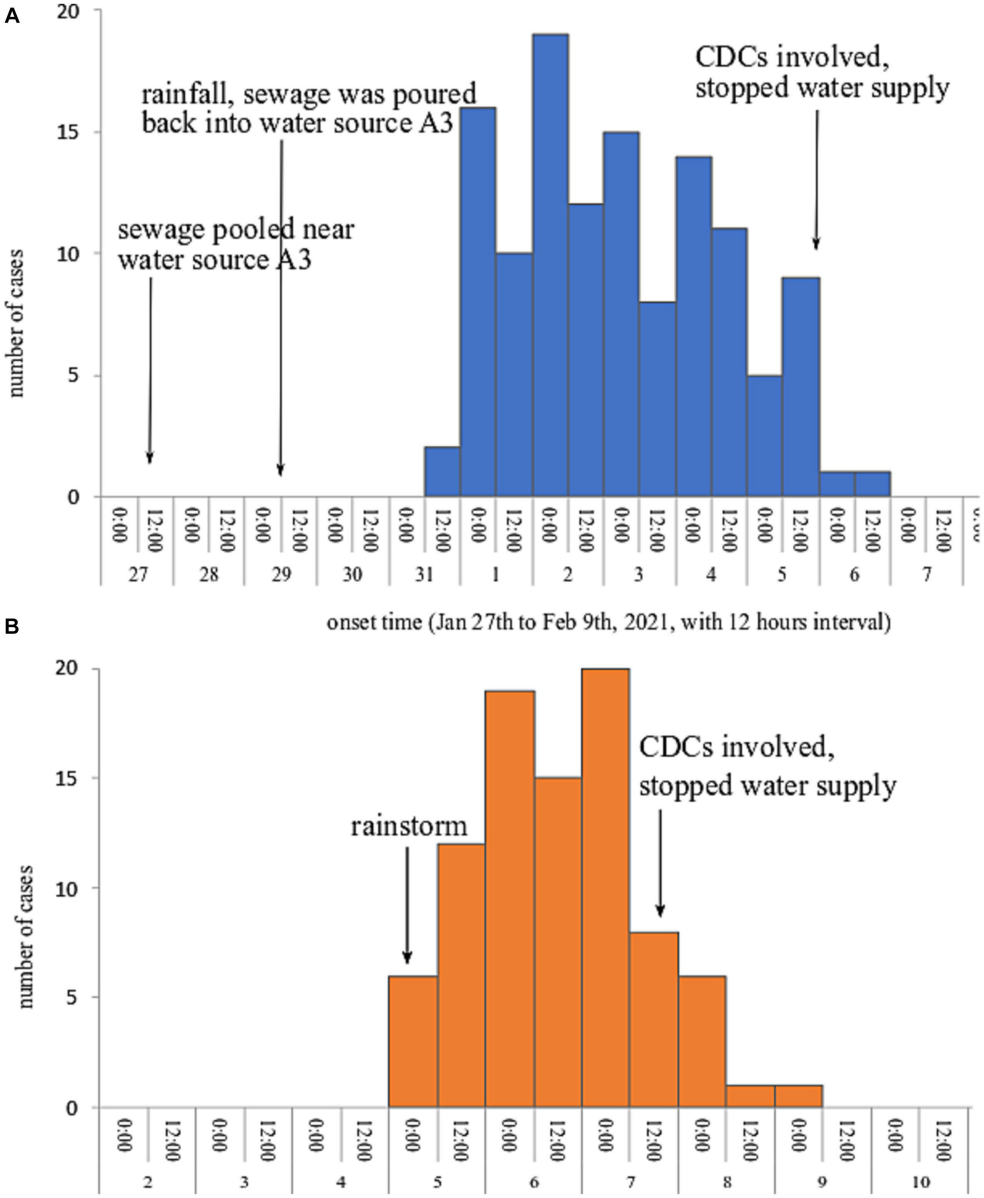
Epidemic curves of the two outbreaks occurred in county A in early 2021.

Both epidemic curves showed a continuous transmission mode, indicating cases exposing continuously to the source of infection. Outbreak A lasted from 31st January to 6th February 2021, the index cases showed symptoms on 31st January and the onset time peaked on February 2, 2021. In outbreak B, the onset time of the first case and the last case was 10:00 on February 5 and 05:30 on February 9, 2021; and the onset time peaked on February 7, 2021. The epidemic curves of the two outbreaks are presented in Figure 1.

### 3.2 Environmental investigation

The two villages use different water sources for their daily life. In outbreak A, there are three spring water sources in the village, water sources A1 and A2 on a hillside and water source A3 located near a creek. These three water sources converge into a large reservoir. In general, water sources A1 and A2 supplied drinking water to local villagers. Water source A3 was reused from January 24, 2021, because a large number of migrant workers returned home leading to unsatisfied water demands. On January 27, the water from the large reservoir was discharged through the ditches to households. Due to a lower altitude of water source A3, sewage overflowed from the ditches and formed large puddles less than one meter away from water source A3. On January 29 and 30, rainfalls caused the sewage from puddles to flow to water source A3. What’s worse, the large reservoir had not been dredged and disinfected for over 10 years.

In outbreak B, there are also three water sources (B1–B3), all of which are spring water sources. The three water sources flow into one reservoir that had not been cleaned and disinfected for over 7 years. Before the occurrence of outbreak B, there was heavy rainfall at midnight on February 4, 2021. The layouts regarding the water sources and water supply systems for the two outbreaks are shown in Supplementary Figure S2.

### 3.3 Retrospective cohort study

A retrospective cohort study was conducted to examine the role of the spring water supply as the outbreak source. Residents in neighboring villages with different water sources served as the unexposed population. In outbreaks A and B, the attack rate of the exposed group was markedly higher than the unexposed group (*p* < 0.001). The results of the retrospective cohort study concluded that contaminated drinking water was the risk factor for both outbreaks. Table 2 shows detailed information.

**Table 2:** Analysis of risk factors of the two norovirus outbreaks.

Contaminated drinking water	Exposed group		Unexposed group		RR	95% CI
	Cases	Total	Cases	Total		
Outbreak A	123	561	0	188	41.85	5.89–297.39
Outbreak B	88	326	0	290	79.23	11.11–565.12

RR, risk ratio; RR was calculated by adding one in each exposure group; CI, confidential interval.

### 3.4 Laboratory detection findings

In outbreak A, a total of nine rectal swabs and one stool specimen from cases, three swab samples from cases’ houses, and six water samples were detected positive for norovirus GII. In outbreak B, a total of 52 samples were detected positive for norovirus GII, including 37 rectal swabs, five stool samples, five swab samples from cases’ houses, and five water samples. The laboratory results are exhibited in Supplementary Tables S1, S2.

### 3.5 Genotyping and polygenetic analysis

In outbreak A, only two rectal swabs have successfully identificated, revealing the presence of two distinct strains: GII.17 (P17) and GII.2 (P16). The GII.17 (P17) strain was highly homologous (99.59%) to a 2016 Brazilian isolate (MH746992.1), while the GII.2 (P16) strain was highly homologous (99.25%) to a 2017 Zhejiang isolate (MH806429.1).

**Figure 2:**
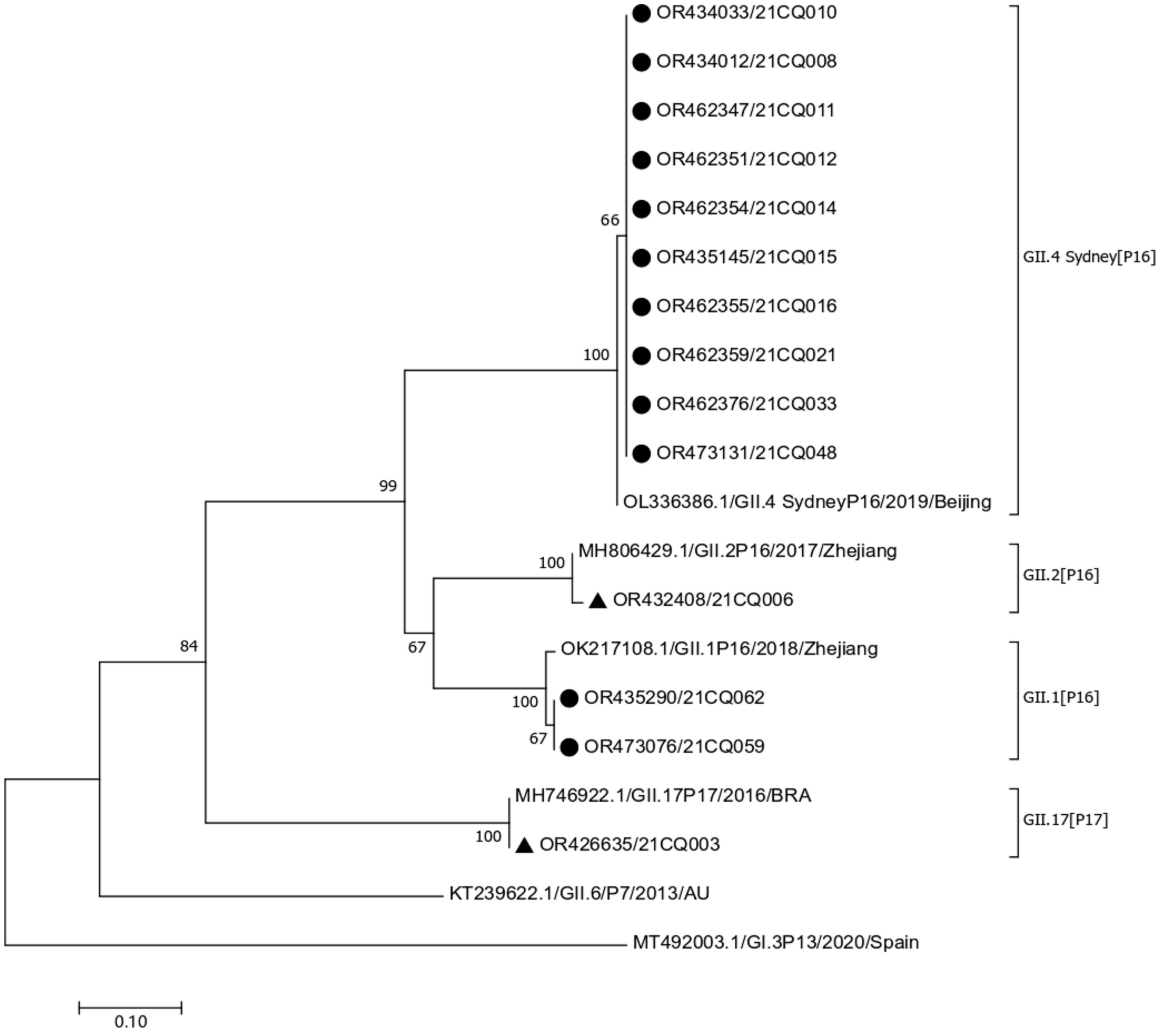
Phylogenetic tree based on partial nucleotide sequences in the polymerase-capsid region. “▲,” sequences obtained from outbreak A, “●,” sequences obtained from outbreak B.

In outbreak B, a total of 16 samples (13 rectal swabs and three stool specimens) revealed the presence of two strains. Fourteen samples (11 rectal swabs and three stool specimens) were identified as GII.4 Sydney (P16) strain, all of which have high homology (99.01%–99.82%) with a 2019 Beijing isolate (OL336386.1). Two rectal swabs were identified as GII.1 (P16) strain which is highly homologous (97.99%–99.10%) to a 2018 Zhejiang isolate (OK217108.1). The phylogenetic tree of the sequences obtained in the two outbreaks is shown in Figure 2.

### 3.6 Control measures

To address the two outbreaks, a series of prompt and effective response measures were conducted. Firstly, the centralized spring water supply in these affected villages was promptly discontinued. Secondly, qualified cleaning and standardized disinfection (with chlorine-containing disinfectant) regarding water sources, reservoirs, and cases’ homes were conducted. Thirdly, an emergency surveillance system for diarrhea cases was activated to detect potential infections in this county. Finally, gatherings, such as playing mahjong and banquets, were minimized. Additionally, health education regarding norovirus during the outbreak response was strengthened, including hand hygiene, the management of feces from humans and animals and cases’ excreta, to prevent contamination of drinking water sources.

## 4 Discussion

Norovirus causes the most outbreaks of non-bacterial gastroenteritis in human beings, which is affected by many climate factors, including low temperature (−6.6°C to 20°C), relative humidity (10% to 66%), and rainfall (1 day to 3 months) (15). Norovirus in groundwater can remain detectable for over 3 years and can remain infectious for at least 61 days (16). In this study, there were rainfalls before the two outbreaks. Thus, rainfalls might lead norovirus in nature to contaminate the spring water sources. Studies have shown that norovirus could persist long in the water environment (17, 18), and cold temperatures could further prolong its viability (19). At the same temperature, the persistence of norovirus was longer in drinking water than in wastewater. During the two outbreaks, the local temperature was below 15°C from January 27 to February 11, 2021, which might extend the persistence of norovirus in drinking water, potentially leading to the emergence of the two outbreaks. The rainfall and low temperature in the two villages contributed to the epidemic of both norovirus outbreaks.

Contaminated drinking water is a common source of norovirus outbreaks among individuals (20, 21). In our study, water samples collected from the water source and cases’ homes both detected positive for norovirus, clearly indicating that drinking water has been contaminated by norovirus. In this remote county, there was poor sanitation, unqualified water purification equipment, and even limited or no access to municipal water. The local villagers had to depend on these natural water sources for their livelihood. Then villagers would be exposed when contaminated water is used for drinking, cooking, or entertainment. These high-risk factors (rainfall, low temperature, poor sanitation, unqualified water purification, and limited access to municipal water) increased the possibility of exposure to contaminated drinking water for villagers.

Our finding revealed that multiple norovirus GII strains are distributed widely in contaminated water sources and implicated cases of different GII strains-associated outbreaks had no significant differences in age and gender. In recent years, GII.4 was the predominant genotype of norovirus gastroenteritis in China, GII.P17, GII.17 strains, and GII.2 had a rapid increase (22–25), also GII.2 (P16) was the main genotype causing norovirus outbreaks from October 2016 to September 2019 (26). Waterborne and person-to-person transmissions were observed in our two outbreaks, which aligns with previous studies that have revealed norovirus GII transmition by food, water (27), or person-to-person contact (24).

In both outbreaks, evidence from epidemiological investigation, environmental investigation, and laboratory detection consistently indicated the epidemiological source and pathogenic etiology. Diarrhea and vomiting are the most common clinical symptoms of norovirus illness, which both were prevalent in both outbreaks. Besides, the epidemiological investigation and environmental investigation indicated that drinking water was the risk factor for each outbreak. Most importantly, PCR tests and sequencing analysis proved that contaminated drinking water was the source of the outbreaks.

Nevertheless, two limitations should not be ignored in this study. First, because of limited laboratory conditions, the concentration of the water sample was completely manual, with a theoretical enrichment of approximately 14-fold, but it had not been verified. Second, owing to the quality of the samples, the water samples in both outbreaks could not be successfully sequenced and were only detected as the GII strains. This limitation cannot directly indicate the connection between contaminated drinking water and cases. It highlights the critical importance of sample quality in future investigations.

In conclusion, the two acute gastroenteritis outbreaks that occurred in a remote county were independent. Both outbreaks were linked to contaminated drinking water and norovirus GII strains. Cases from different GII strains-associated outbreaks showed no significant differences in age, gender, and occupation. It revealed potential safety hazards of spring water supply in this rural county, reinforcing the importance of good sanitation (hand hygiene, the management of feces from humans and animals and cases’ excreta) and environmental disinfection (regular water source disinfection) in rural areas, especially during the rainy seasons.

## Data availability statement

The datasets presented in this study can be found in online repositories. The names of the repository/repositories and accession number(s) can be found in the article/Supplementary material.

## Ethics statement

As an immediate response to a public health emergency, investigations were initiated upon the occurrence of an outbreak. In accordance with Article 12 of Chapter I of the Law of the People’s Republic of China on the Prevention and Control of Infectious Diseases (28) (available at: http://en.nhc.gov.cn/2019-03/05/c_74526.htm), all related units and individuals must accept the investigation, inspection, and sample collection by the investigation offices and provide relevant information truthfully. Therefore, ethical approval and participant consent were exempted from these investigations for public health emergency response.

All samples and data collected from participants were used exclusively for this study, with investigators ensuring the confidentiality of participants’ responses and the sample data. Data analyses were conducted anonymously, without disclosing any personal information about the participants. Research involving human participants, human material, and human data had been performed under the Declaration of Helsinki.

## Author contributions

TL: Data curation, Formal analysis, Investigation, Visualization, Writing – original draft, Writing – review & editing. JP: Data curation, Funding acquisition, Methodology, Visualization, Writing – review & editing. QL: Conceptualization, Methodology, Project administration, Resources, Writing – review & editing. BL: Data curation, Formal analysis, Investigation, Writing – review & editing. YY: Data curation, Investigation, Visualization, Writing – review & editing. CY: Data curation, Investigation, Resources, Writing – original draft, Writing – review & editing. DY: Data curation, Formal analysis, Investigation, Writing – original draft, Writing – review & editing. WT: Conceptualization, Investigation, Project administration, Resources, Writing – review & editing. LQ: Conceptualization, Formal analysis, Funding acquisition, Investigation, Writing – original draft, Writing – review & editing.

## Footnotes

^1^https://www.ncbi.nlm.nih.gov/

^2^https://www.megasoftware.net

## Supplementary material

### Supplementary Figure S1

Laboratory results of samples collected from two outbreaks.

### Supplementary Figure S2

Dataset used in this study.

### Supplementary TABLE S1

Location of villages for the two outbreaks occurred.

### Supplementary TABLE S2

Layout of water resources with potential contaminant risks of the two outbreaks.
