# Supplementary figures and images for: Investigation of two norovirus outbreaks linked to drinking water contaminated with multiple GII strains in a rural county—Chongqing, China, 2021
Source: Front Public Health. 2023 Dec 14;11:1259584. doi: 10.3389/fpubh.2023.1259584 (PMC10756231; doi:10.3389/fpubh.2023.1259584)

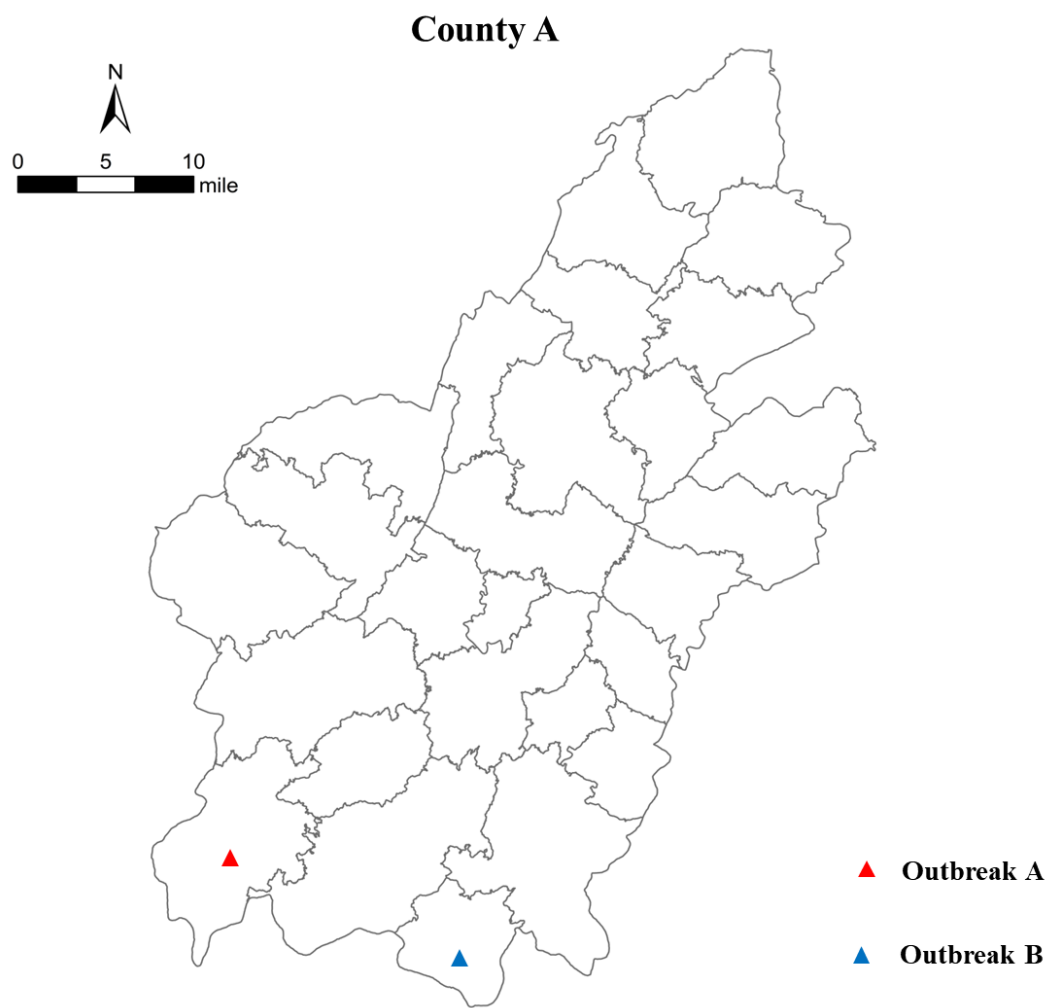

**Supplemenry Figure 1. The location of villages for the two outbreaks occurred.**

Supplement: Supplementary file 1 [file Image_1.pdf]
