# Supplementary material for: Investigation of two norovirus outbreaks linked to drinking water contaminated with multiple GII strains in a rural county—Chongqing, China, 2021
Source: Front Public Health. 2023 Dec 14;11:1259584. doi: 10.3389/fpubh.2023.1259584 (PMC10756231; doi:10.3389/fpubh.2023.1259584)
Supplement: Supplementary file 3 [file Table_1.DOCX]

**Supplementary Table 1**

**Laboratory results of samples collected from two outbreaks.**

| **Samples** | **Outbreak A (**pos./test, %**)** | **Outbreak B (**pos./test, %**)** |
| --- | --- | --- |
| Cases’ samples | 10/14, 71.43 | 42/50, 84.00 |
| Anal swab | 9/13, 69.23 | 37/44, 84.09 |
| Stool sample | 1/1, 100 | 5/6, 83.33 |
| Samples from cases’ house | 3/8, 37.50 | 5/11, 45.45 |
| Water tank | 1/3, 33.3 | 0/1, 0 |
| Water tap | 2/4, 50.00 | 3/5, 60 |
| Water tank+ Water tap | 0/1, 0 | 2/4, 50 |
| Other samples from cases’ houses* | — | 0/1, 0 |
| Water samples from water sources | 6/7, 85.71 | 5/11, 45.45 |
| Reservoir water | 1/2, 50.00 | 1/2, 50.00 |
| Water source^#^ | 3/3, 100 | 4/9, 44.44 |
| Terminal tap water | 1/1, 100 | — |
| Puddle of water near water source A3 | 1/1, 100 | — |

Note: pos., number of positive samples. “—” indicates no samples collected. *, the smear sample of the inner wall of the pool for washing vegetables. ^#^, in outbreak A, water samples were collected from water source A3; in outbreak B, six water samples were collected from water source B1–B3, and three water samples were collected from another water source supplied for the villagers in another village (all samples from another village tested for norovirus negative).
